# Design of a Biocatalytic Filter for the Degradation of Diclofenac and Its Ozonation Products

**DOI:** 10.1002/elsc.70024

**Published:** 2025-05-02

**Authors:** Dorothee Schmiemann, Jessica Schneider, Marcel Remek, Jeremy Kaulertz, Oliver Seifert, Monika Weidmann, Klaus Opwis, Arno Cordes, Martin Jäger, Jochen Stefan Gutmann, Kerstin Hoffmann‐Jacobsen

**Affiliations:** ^1^ Department of Chemistry and Institute for Coatings and Surface Chemistry Niederrhein University of Applied Sciences Krefeld Germany; ^2^ Institute of Physical Chemistry and CENIDE (Center for Nanointegration) University Duisburg‐Essen Essen Germany; ^3^ Deutsches Textilforschungszentrum Nord‐West gGmbH Krefeld Germany; ^4^ ASA Spezialenzyme GmbH Wolfenbüttel Germany

**Keywords:** filter design, immobilized laccase, micropollutant, textile, wastewater treatment

## Abstract

Posttreatment of the effluents from wastewater treatment plants is becoming increasingly important, as the conventional treatment cannot completely remove organic trace contaminants. Promising techniques like chemical oxidation methods, including ozonation, face the challenge of potentially generating more toxic transformation products than their parent substances due to incomplete oxidation. In this work, the laccase from *Trametes versicolor* was immobilized on a polyester textile to create a biocatalytic textile filter for the posttreatment of organic trace contaminants and their ozonation by‐products. Different filter designs for reactive filtration with biocatalytic textiles were implemented on the laboratory scale and tested for their effectiveness in degrading the dye Remazol Brilliant Blue, the pharmaceutical diclofenac, and its ozonation products. The plate module, inspired by lamellar clarifiers and featuring the textile with covalently immobilized enzyme on the lamella surfaces, exhibited the best performance characteristics. Employing this module, a continuous process of diclofenac ozonation and subsequent posttreatment with the biocatalytic filter was conducted. This not only demonstrated the feasibility of continuous biocatalytic wastewater filtration but also highlighted improved degradation efficiencies of ozonation products compared to the batch process using laccase in solution.

**Abbreviations**
BT: breakthrough
DF: diclofenac
LOQ: Limit of Quantification
PAA: polyacrylic acid
PET: polyethylene terephthalate
PM: plate module
RBB: Remazol Brilliant Blue
TP: transformation product
WM: wound module

## 1 Introduction

In recent years, the importance of wastewater posttreatment has grown as conventional wastewater treatment plants have proven unable to completely remove organic trace contaminants from wastewater, including pharmaceuticals, personal care products, dyes, and pesticides [1, 2]. Diclofenac is an important indicator compound, as the nonsteroidal anti‐inflammatory drug is detected world‐wide in most urban wastewaters [3]. Extensive research has been conducted in the field of wastewater treatment, and various chemical and physical methods have already been tested and proven to be effective [4, 5]. These methods include ozonation, advanced oxidation processes (AOPs), the Fenton process, adsorption, and membrane filtration, all aimed to remove pollutants from wastewater [6, 7, 8].

**Summary**

• The removal of micropollutants is a major challenge in the further development of municipal sewage treatment plants.

• The current study presents the realization of a new method combining enzymativ post‐treatment with ozonation for the degradation of micropollutants.

• A continuous enzymatic treatment process is realized by immobilizing a laccase on a technical PET textile and developing filter modules thereof.

• With these filter modules, biocatalytic filtration can be used for the treatment of micropollutants and, in particular, for the posttreatment of ozonation.

• As the biocatalytic filtration modules are developed in accordance with wastewater treatment solutions, the method presented on a laboratory scale is in principle transferable to a larger scale.

Ozonation has been demonstrated to be an effective technique for the post‐treatment of wastewater, from pilot plant to technical scale applications [10, 11]. Nevertheless, it is important to note that chemical processes such as ozonation have the potential to be environmentally problematic, as they can generate transformation products that are more toxic than the original substances. [12, 13]. It is imperative that research be conducted into more sustainable and environmentally friendly methods to ensure the removal of potential transformation products.

Biocatalysts respectively enzymes, characterized by their high efficiency and excellent chemo‐, stereo‐, and regioselectivity, are particularly well suited for this purpose. In addition, enzymes operate effectively over a wide pH and temperature range and do not necessitate the use of high pressures or temperatures. Oxidoreductases are a key class of enzymes studied in wastewater engineering, due to their efficacy in the removal of dyes and trace substances. The enzyme laccase is one of the best‐known examples [14, 15, 16]. Using molecular oxygen as an electron acceptor, laccases catalyze the oxidation of various phenols and aromatic or aliphatic amines to the corresponding radicals, optimally leading to oligomers, which exhibit lower toxicity [17, 18]. We previously demonstrated that laccase from *Trametes versicolor* is not only capable of degrading several micropollutants, such as diclofenac, but is particularly effective at removing potentially harmful transformation products resulting from micropollutant ozonation [19].

For technical applications, enzymes are typically immobilized on solid supports, producing heterogeneous and reusable biocatalysts. This approach not only offers economic advantages but also enhances enzyme stability across a wide range of temperatures and pH levels, while increasing mechanical resistance [20, 21, 22]. There are many different methods for enzyme immobilization, including adsorptive and covalent methods. Adsorptive immobilization offers the advantage of simple and inexpensive adsorption of enzymes to the surface of a support by hydrogen bonding, van der Waals forces, or ionic interactions, while largely preserving the enzyme conformation. However, the disadvantage of this immobilization is enzyme leaching due to the weak binding [20, 21]. In covalent immobilization, chemical bonds between functional groups of the enzyme and the support are formed with the aid of a crosslinker, and this scaffold can, in turn, be crosslinked. Covalent immobilization typically reduces enzyme leaching but exposes the enzyme to stronger conformational stress, which can also result in a reduction in the activity of the heterogeneous biocatalyst [20, 21].

A broad variety of material has been used as supports ranging from inorganic particles, inorganic‐organic scaffolds, and organic resins [23, 24]. Textiles have been used previously for the immobilization of the oxidoreductases catalase and peroxidase [25, 26, 27]. The immobilization of laccase on different carriers, such as electrospun nanofibers, carbon nanotubes, nanofiber membrane, polyamide/polyethyleneimine fibers, micro‐biochar, granulated activated carbon, alumina spherical pellets, ultraporous alumina and corn cob for the degradation of micropollutants, such as diclofenac [28, 29, 30, 31, 32, 33, 34, 35] and RBB [36, 37, 38] in batch processes has also been described as the posttreatment of combustion processes [39]. Continuous processes have been successfully implemented using either dissolved or immobilized enzymes in membrane reactors [40, 41, 42, 43].

This study aims to develop a continuous method for the removal of trace substances and their ozonation products from wastewater using a biocatalytic textile, generated by immobilizing the laccase from *T.s versicolor* on a polyester needle felt. Different immobilization methods, that is, adsorptive and covalent immobilization, as well as textile filter set‐ups are analyzed on a laboratory scale using the degradation of the dye RBB and the pharmaceutical diclofenac as model trace contaminants.

The wound module exerts a force upon the water, directing it through the biocatalytic textile. Meanwhile, the plate module deploys the fabric on the plates of a lamella‐type clarifier, thereby directing the water over the textile surface. The analysis of the breakthrough curves of the continuous degradation of the model trace contaminants are used to identify the best filter design. This set‐up is then applied to the degradation of diclofenac and its transformation products in a continuous chemo‐enzymatic process using ozonation and subsequent enzymatic post‐treatment, which is analyzed by HPLC‐HRMS (High Performance Liquid Chromatography‐High Resolution Mass Spectrometry).

## 2 Materials and Methods

### 2.1 Materials

Diclofenac sodium salt (DF, 98%) was purchased from Alfa Aesar (Kandel, Germany). Remazol Brilliant Blue BB (RBB 220), with CAS number 128416‐19‐3, was an in‐house sample. The polyester (PET) felt used (Type E20102 0/G) was supplied by Kayser Filtertech GmbH (Einbeck, Germany). The polyacrylic acid (PAA) DEGAPAS 4104 S was procured from Evonik Industries AG (Essen, Germany). The hexadiisocyanate crosslinker Bayhydur XP 2487/1 was purchased from Covestro (Leverkusen, Germany). Acetonitrile (ACN) (LC‐MS Grade), bovine serum albumin (BSA) Fraction V, Coomassie Brilliant Blue G‐250, ethanol (≥99.50) and ortho‐phosphoric acid (85%) were purchased from Carl Roth (Karlsruhe, Germany). Sodium hydroxide solution (1 mol/L) was purchased from Bernd Kraft. Formic acid (LC‐MS grade) was purchased from Fluka‐Honeywell (Seelze, Germany). Syringaldazine for laccase activity measurement was purchased from Sigma Aldrich (Steinheim, Germany). The laccase from *T. versicolor* was obtained from ASA Spezialenzyme GmbH (Wolfenbüttel, Germany). For solid phase extraction (SPE), Oasis HLB 3 cc cartridges (60 mg sorbent per cartridge, particle size 30 µm) from Waters (Eschborn, Germany) were applied.

### 2.2 Enzyme Immobilization

The polyacrylic acid (PAA) modified PET felt was obtained from Grenzland (Bocholt, Germany). The coating solution consisted of 50% Degapas 4104S (PAA, 40% purity), 5% Bayhydur, and 45% deionized water. The polyester textile was prepared on a Coatema machine and thermally fixed at 170°C to achieve a final weight of approximately 65%. The pre‐functionalized textile was treated with an aqueous enzyme solution (Laccase *T. versicolor*, 30 g/L). The textile absorbed 3.3 mL/g net weight. After enzyme treatment, the textile was dried overnight at room temperature.

For covalent immobilization, a white homogeneous mixture of 6% w/v PAA (Degapas 4104S) and 4% w/v Bayhydur in deionized water (resulting in a pH of approximately 2.5) was prepared. The enzyme powder was added while stirring slowly to obtain a concentration of 30 g/L, resulting in a pH value of approximately 4.1.

After no more than 1 h, the mixture dissolved, and the solution was ready to be applied to the textile and mechanically worked into the fabric. The textiles were dried at room temperature at least 24 h. Both the adsorptive and covalent textiles were stored at 6°C in a refrigerator.

### 2.3 Analysis

Immobilized laccase activity was measured before and after each degradation experiment using the syringaldazine assay (530 nm) at pH 5 with a 7.7 µM syringaldazine solution in *d*H_2_O at 20°C as described previously [44].

The protein content of the biocatalytic textiles was determined with a Bradford assay. Textile pieces (ca. 50 mg) were introduced into 0.1 mmol/L NaOH solution, agitated for 1 min, and incubated for 24 h. After short agitation the supernatant was removed from the fabrics by centrifugation and 100 µL of the supernatant were analyzed with the Bradford assay detecting the absorbance at 595 nm (UV‐Vis spectrometer, Hach Lange). The successful immobilization of the laccase on the surface of the PET textile was qualitatively analyzed by UV‐Vis spectroscopy (Lambda 950 S, Perkin Elmer) using an integrating sphere (200–800 nm). Scanning electron micrographs of the fabrics were taken with a Hitachi S‐3400N microscope [25].

The concentration of RBB was analyzed via the absorption at 605 nm with a UV‐1650 PC spectrophotometer (Shimadzu, Duisburg, Germany). The LOQ was 1.10 mg/L. Diclofenac concentrations were analyzed by reserved phase chromatography using an Agilent 1260 Infinity II series high performance liquid chromatography (HPLC) system (Agilent Technologies, Inc., Waldbronn, Germany) equipped with a Poroshell 120 EC‐C18 (2.7 µm, 4.6 × 100 mm, Agilent Technologies, Inc., Waldbronn, Germany) and UV detection (275 nm). The eluents were *dd*H_2_O (A) and ACN (B), each containing 0.1% formic acid. The flow rate was 0.3 mL/min, the column temperature was 40°C, and the injection volume was 5 µL. The LOQ of diclofenac with the LC‐UV method was 175 µg/L. The ozonation products of diclofenac were analyzed by HPLC‐HRMS. The LC‐MS/MS system consisted of an Agilent 1200 series HPLC system (Agilent Technologies, Inc., Waldbronn, Germany) coupled to an Agilent 6530 Q‐ToF mass spectrometer (Agilent Technologies, Santa Clara, USA) with jet‐stream electrospray ion source (ESI) (negative mode, fragmentor voltage 125 V) using the column Eclipse Plus C18 (ZORBAX, 3.5 µm, 2.1 × 150 mm, Agilent Technologies, Inc., Waldbronn, Germany) as described previously [44]. Samples were concentrated 20‐fold by SPE.

### 2.4 Filter Set‐up

The CAD designs of the plate and wound module are depicted in Figure 4. The basin (25 cm × 8 cm × 12.5 cm) for the plate module was built from 6 mm thick acrylic glass plates, which were glued with acrylic adhesive (ACRIFIX 1R 0192, Röhm GmbH, Darmstadt, Germany). The lamellar inset was created using a 3D printer (UltiMaker S5, Ultimaker B.V., Geldermalsen, Netherlands) and polylactic acid as material (Ultimaker PLA Transparent, Ultimaker B.V., Geldermalsen, Netherlands). It consisted of a rail for five textile‐covered plates (11.2 cm × 7.5 cm). Following the design of a parallel plate separator the plate module was designed with an inclination of 55° [45]. To avoid shortcuts, a stirring fish was inserted under the first two textile‐covered plates in the PM.

The core of the wound module was constructed from steel tubings and fittings of heating technology (Heinrich Schmidt GmbH & Co. KG, Mönchenglabach, Germany): two 3/8″ tube sockets connected by a sleeve, a double tube containing twelve 6 mm holes, and a 3/8″ and 1″, respectively, reducing sleeve at the ends. The 3/8’’ sleeve was plugged to prevent the fluid from leaking away from the holes. The other side was open, and a rubber seal was glued onto the reducing sleeve. The textile was wrapped around the double tubes. The ends were sealed with reducing sleeves, a rubber seal and silicone (Sista F101, Henkel AG & Co. KGaA, Düsseldorf, Germany) and introduced into a preparative chromatography column (ECO50/450M3V, YMC Europe GmbH, Dinslaken, Germany) (Figure 4).

### 2.5 Breakthrough Analysis

Pollution degradation in the textile filter modules was performed with 840 cm^2^ filter area in each experiment. The reaction volume in the plate module was 826 and 413 mL in the wound module, respectively. The modules were fed with a peristaltic pump (Ismatec BVP ISM444B, Glattbrugg, Switzerland). The process conditions were chosen such that the mean residence time was 2.75 h for both modules. Accordingly, the flow rate was 5 mL/min for the plate module and 2.5 mL/min for the wound module, respectively.

All experiments were conducted at room temperature (23°C ± 2°C). The initial concentration of RBB was 68.63 ± 0.82 mg/L and of diclofenac 10.67 ± 0.76 mg/L. Ozonation was performed as described previously [44]. A 20 mg/L diclofenac solution was ozonated for 6 min with a capacity of 10 g O_3_/m^3^ and subjected to posttreatment in the plate module.

The breakthrough curves were calculated using the Gompertz function (Equation 1) [46].

(1) $$y=a\cdot e^{-e^{\left(-k\left(x-BT\right)\right)}}$$

with *x* being the independent variable, *a* the amplitude or the initial concentration of the respective substance, *k* is a coefficient describing the slope and *BT* represents the breakthrough point of the curve.

## 3 Results and Discussion

### 3.1 Characterization of the Biocatalytic Textiles

**FIGURE 1:**
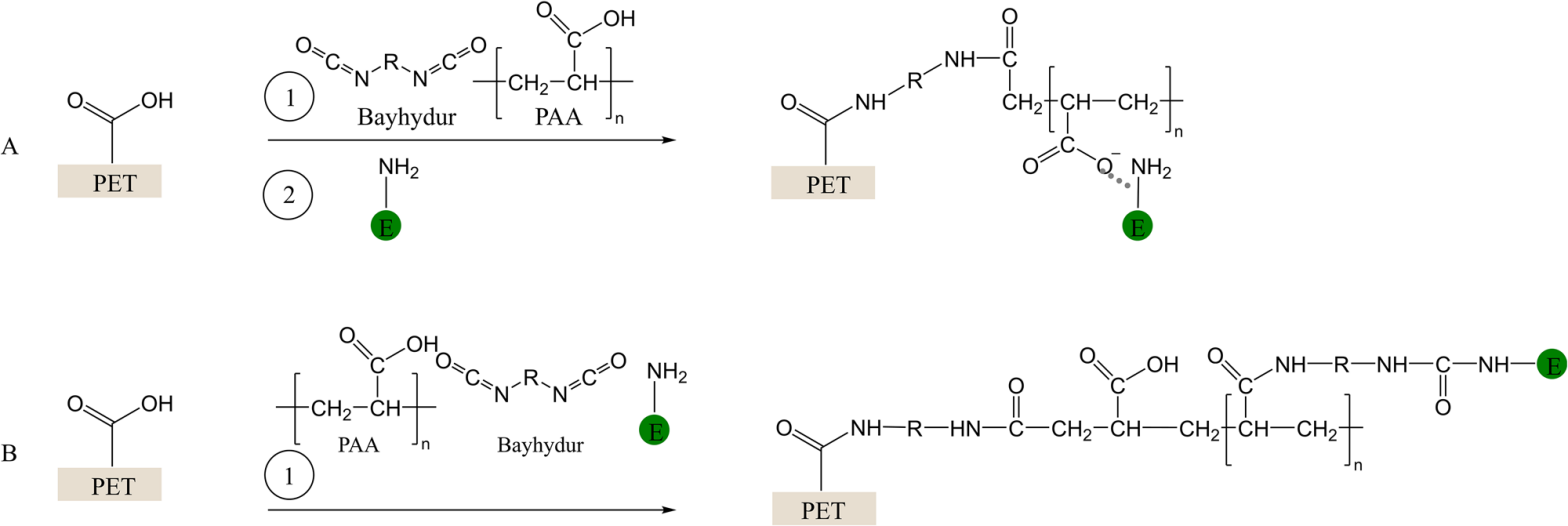
Proposed reaction mechanism of adsorptive (A) and covalent immobilization (B) of the laccase from *T. versicolor* [E].

Figure 1 illustrates the reaction scheme for both adsorptive and covalent immobilization of laccase on PET. For adsorptive immobilization, the isocyanate crosslinker (Bayhydur) and PAA are initially thermally fixed onto the PET to generate carboxylate groups on the PET surface, where adsorption of the enzyme takes place. For covalent immobilization, PAA, the isocyanate crosslinker and the enzyme are blended in a one‐pot preparation and subsequently applied to the unfunctionalized PET textile. In this case, various covalent bonds, potentially carbamate bonds, between the enzyme, PAA and the textile are formed, which lead to enzyme immobilization as well as crosslinking.

**FIGURE 2:**
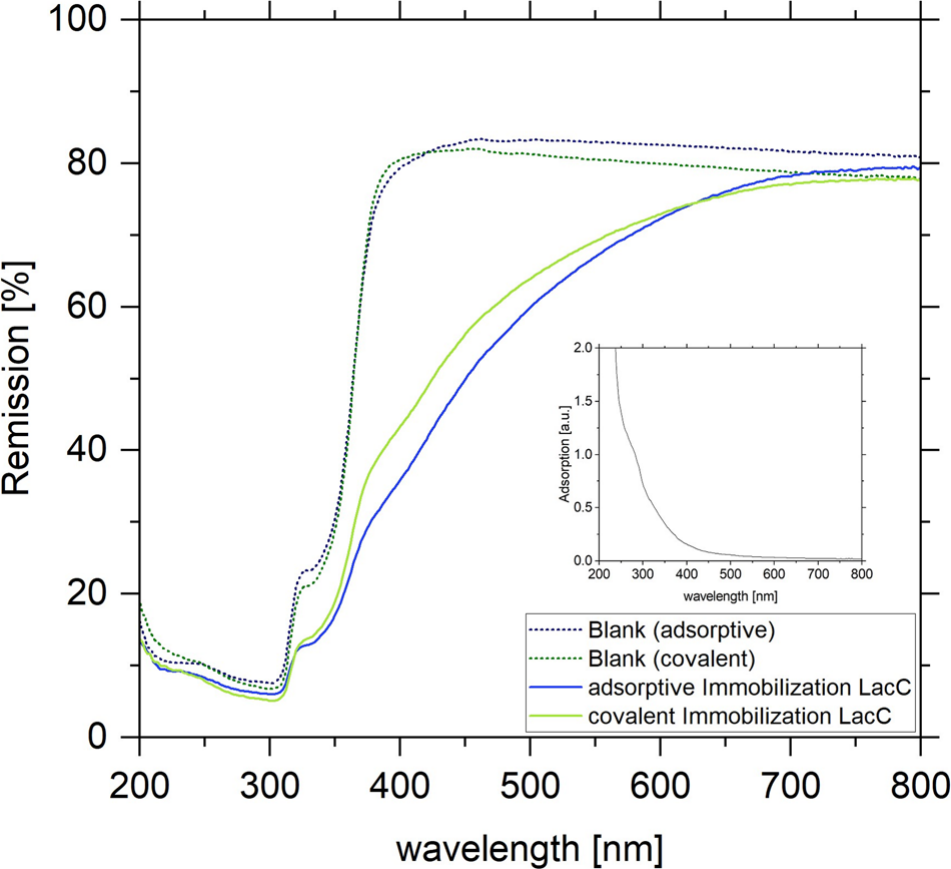
UV‐Vis remission spectrum of textiles before and after adsorptive (blue) and covalent (green) immobilization of laccase *T. versicolor*. The inset provides the absorbance spectrum of laccase of *T. versicolor*.

The qualitative analysis of the immobilization of the laccase was conducted using UV‐Vis remission spectroscopy. Figure 2 depicts the UV‐Vis spectra of the PET textile with and without laccase. The application of both immobilization techniques resulted in a reduction in diffuse reflectance at 400 nm, which is indicative of the presence of laccase on the textile.

**FIGURE 3:**
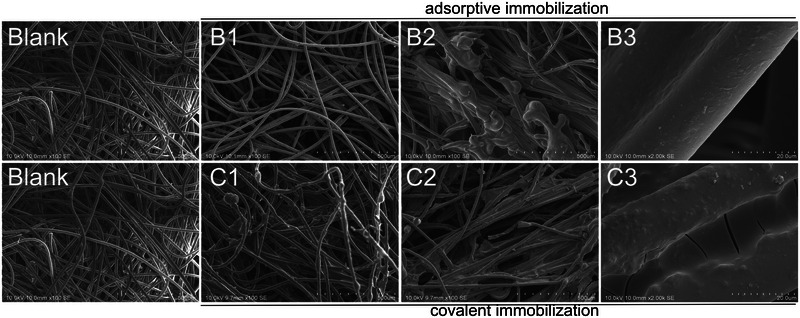
SEM images of adsorptive (B2–B3) and covalent (C2–C3) immobilization of laccase *T. versicolor*. Blank: untreated polyester needle felt, B1: PET pre‐functionalized with Bayhydur and polyacrylic acid for adsorptive immobilization. B2 and B3: PET after adsorptive immobilization of the laccase at 100× and 2000× resolution, respectively. C1: PET treated with one‐pot PAA/Bayhydur solution without enzyme. C2 and C3: PET after covalent immobilization of laccase at 100× and 2000× resolution, respectively.

The process of enzyme immobilization can be observed and analyzed through the use of electron microscopy. Figure 3 depicts the scanning electron micrographs of the distinct phases of immobilization for the adsorptive (first row) and covalent (second row) method. The pre‐functionalization of the textile with Bayhydur and PAA resulted in the formation of a thin but visible coating layer on and between the fibers (B1). Following the adsorptive immobilization of laccase (B2–B3), the formation of thicker attachments to the single fibers and within the interstitial spaces was observed. To serve as a point of comparison for the covalent immobilization process, a one‐pot procedure was conducted without the enzyme (C1). The resulting electron micrograph revealed a coating of the fibers, which was thicker and less homogeneous than in the case of large‐scale mechanical coating (B1). Following the application of the enzyme with the polymer and crosslinker solution for covalent immobilization, a dense coating of the textile was evident, particularly in the interstitial spaces between the fibers (C2–C3).

The textile biocatalyst was subsequently subjected to further characterization based on the initial activity and protein load. The protein load was determined by a Bradford assay. As summarized in Table 1, both immobilization methods yielded comparable initial properties of the biocatalytic textiles. It should be noted that the activity analysis of the biocatalytic textile was affected by the strong adsorption of the oxidized syringaldazine on the textile surfaces. This introduced a systematic error into the activity data, the extent of which is not quantifiable.

**TABLE 1:** Activity of the adsorptive and covalent immobilized laccase *T. versicolor* during the start of the experiments, as well as the protein concentration according to Bradford.

Immobilization method	Activity (U/g)	Protein (mg/g)
Adsorptive	10.3 ± 0.57	0.69 ± 0.08
Covalent	10.6 ± 1.20	0.98 ± 0.30

### 3.2 Evaluation of the Different Filter Designs via Degradation of the Dye Remazol Brilliant Blue

**FIGURE 4:**
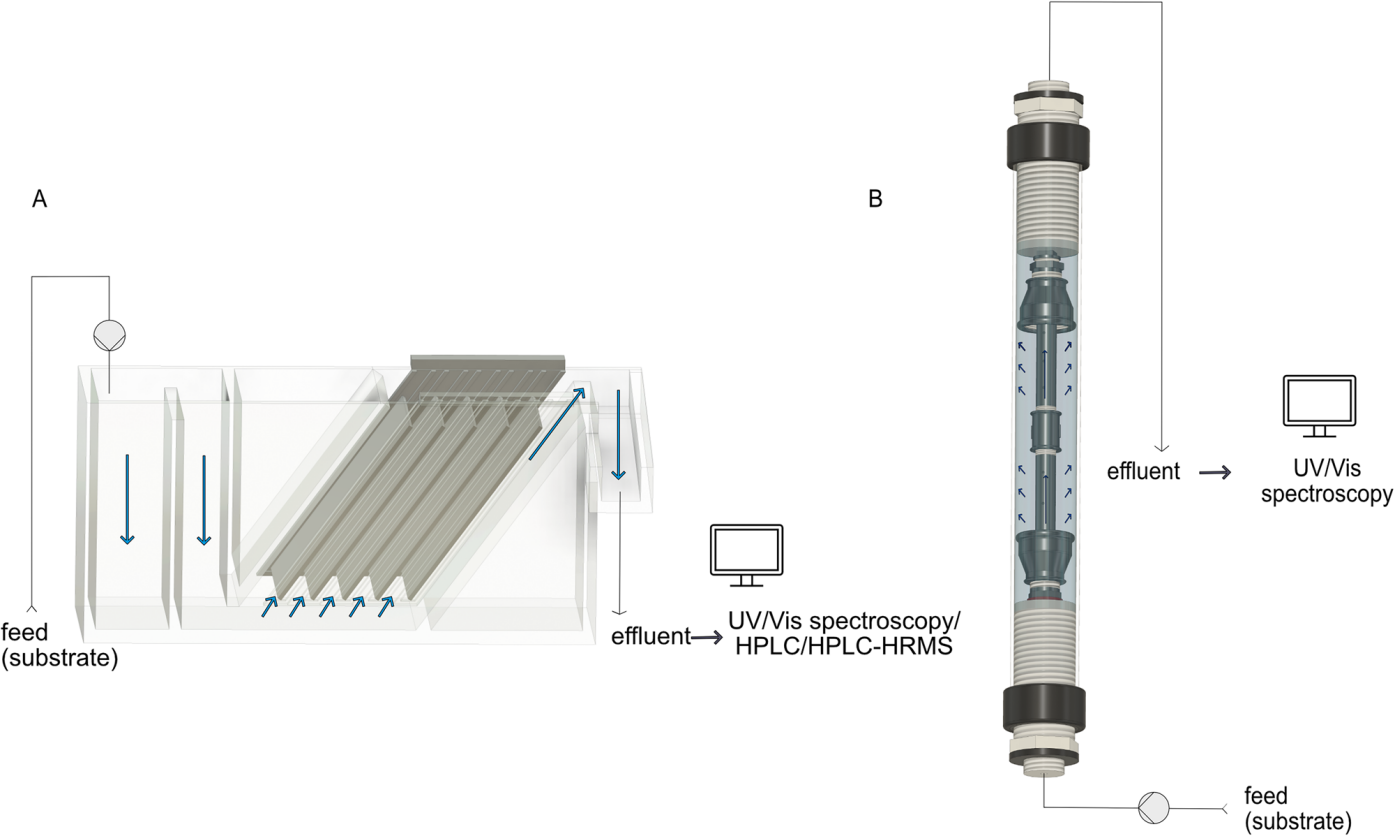
Design of the enzymatic filter in the form of a plate module (A) and a wound module (B) in the continuous process.

Two contrasting designs for a biocatalytic filter utilizing the textiles equipped with laccase were developed (Figure 4). In the plate module (PM), the fabric was used to enclose lamella plates within a basin that was designed in accordance with the conventional configuration of lamella clarifiers. In this configuration, the water was guided along the surface of the textile. The design of the wound module (WM) was guided by the principles of membrane technology. The textile was wrapped around a perforated flow tube. Thus, the water was forced to flow through the biocatalytic textile.

In the first step, the performance of the two different filter types was evaluated using the anthraquinone dye, Remazol Brilliant Blue (RBB), as model pollutant which is known to be decolorized by laccases. A continuous flow of RBB solution (69 mg/L) was treated in the plate and wound module and the breakthrough curves of the respective filter equipped with adsorptively and covalently bound laccase were analyzed for comparison. The filters were constructed with identical filter areas, measuring 840 cm^2^. The flow rate was calibrated to correspond with the varying filter volumes, thereby ensuring that the residence times remained consistent at 2.75 h.

**FIGURE 5:**
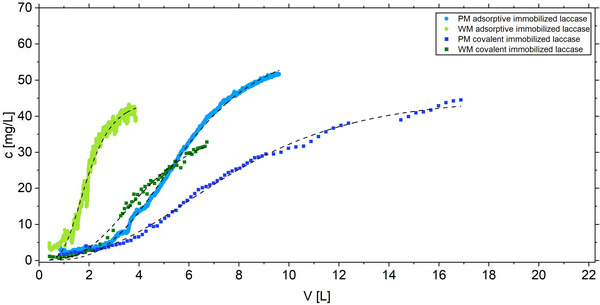
Degradation of RBB in the plate module (blue) and wound module (green) by adsorptive and covalently immobilized laccase *T. versicolor* at RT. The black dashed lines represent the fit according to the Gompertz function.

The breakthrough curves were fitted to Equation (1). As illustrated in Figure 5 and Table 2, the breakthrough of the enzymatic filter shifted to larger volumes in both designs when covalent immobilization was used. In the WM, the service life until breakthrough was found to be twice as long when employing covalent immobilization instead of adsorptive immobilization. In contrast to a conventional filter, the breakthrough was attributed to a decline in biocatalytic activity rather than the saturation of the filter. The decline in activity at an early stage, observed in the case of adsorptive binding of the enzyme, was attributed to leaching of the enzyme. The increased durability of the biocatalytic textile prepared via covalent enzyme adsorption indicated that the isocyanate crosslinker reinforced enzyme immobilization, resulting in augmented irreversible enzyme attachment. After covalent immobilization, the cooperativity of the breakthrough was reduced as reflected in the smaller *k* (Table 2), in contrast to the textiles with adsorptive bound enzyme.

**TABLE 2:** Numerical analysis with Equation (1) of the continuous RBB removal using a biocatalytic textile obtained from the indicated immobilization method in the wound (WM) and the plate module (PM): The breakthrough point (BT) is given in terms of flow volume and operation time to breakthrough, *k* is the slope parameter from the Gompertz function, *removal* depicts RBB removal until breakpoint, and *N_V_* the number of column volumes until BT.

	Immobilization	*k* (L^−1^)	*BT* (L)	*BT* (h)	Removal (%)	*N_V_*
WM	Adsorptive	1.42 ± 0.02	1.64 ± 0.01	5.47 ± 0.03	91.2 ± 3.1	3.97 ± 0.02
	Covalent	0.58 ± 0.03	3.45 ± 0.06	11.50 ± 0.2	94.5 ± 0.4	8.35 ± 0.15
PM	Adsorptive	0.44 ± 0.002	4.88 ± 0.01	16.27 ± 0.03	89.8 ± 2.7	5.91 ± 0.01
	Covalent	0.27 ± 0.006	5.99 ± 0.06	19.97 ± 0.2	91.4 ± 0.4	7.25 ± 0.01

This indicated that covalent immobilization achieved persistent activity from irreversibly bound enzymes on the textile, even after reaching the numerically determined breakthrough points. However, it is also possible that enzyme damage may contribute to the reduction in biocatalytic activity with increasing operation time. This hypothesis is supported by the observed decrease in activity over time during enzyme membrane reactor operation [41]. The residual activity of the textiles after use in the PM module was 0.51 ± 0.09 U/g in the case of covalent immobilization, while the textile equipped via enzyme adsorption exhibited near‐complete depletion (0.03 ± 0.002 U/g). The residual activity of the covalently finished textile was expected to be even greater, due to the intense pink color of the covalently immobilized textile indicating the adsorption of converted syringaldazine after the activity assay (Figure S1).

In reference experiments without enzyme, the textile which was mechanically equipped with PAA and Bayhydur (Figure 3, B1) was used. Here, no RBB adsorption was observed (Figure S2A). This proved that the RBB removal illustrated in Figure 5 was entirely of biocatalytic nature. In contrast, the less homogeneous surface obtained by finishing the textile with PAA/isocyanate using the one‐pot method (Figure 3, C1), led to detectable RBB adsorption. Over the test period, RBB adsorption of 9.1% and 25% were observed for PM and the WM, respectively (Figure S2B). This indicated that the RBB removal observed specifically in the WM using covalent immobilization could not be fully attributed to biocatalytic activity.

A comparison of the breakthrough curves of the two filter designs (Table 2) demonstrated that the plate module was capable of treating a greater volume of water until breakthrough than the wound module. The number of column (or basin) volumes to breakthrough were similar in both modules for the covalent immobilization, but a lower number of column volumes could be treated in the WM than in the PM using adsorptive immobilization. This indicated that passing the water through the filter was not necessary for an efficient biocatalytic treatment of the water. On the contrary, the lower shear forces in the PM favored this design as the performance time of the biocatalytic filter was limited by enzyme leaching. Finally, the basin volume per textile area was larger in the case of the PM, resulting in significantly larger volumes of water that could be treated in the PM than in the WM. Consequently, the PM was employed in the subsequent experiments.

### 3.3 Degradation of Diclofenac in the Plate Module

**FIGURE 6:**
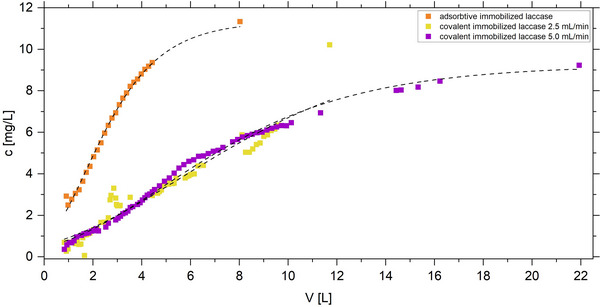
Degradation of DF in the plate module by adsorptive (orange) and covalently (purple and yellow) immobilized laccase *T. versicolor* at different flow rates, yellow: 2.5 mL/min, purple: 5.0 mL/min. The black dashed lines represent the fit according to the Gompertz function.

The potential of a biocatalytic textile filter for the degradation of micropollutants was analyzed using the drug diclofenac (DF) as a model compound based on degradation experiments with RBB dye. Compared to the degradation of RBB, the degradation of 10 mg/L DF exhibited a less symmetrical breakthrough curve (Figure 6). Especially when using adsorptive immobilization, no plateau was observed at the beginning of the experiment indicating continuous leaching of the enzyme. The covalently bound enzyme demonstrated superior performance in DF degradation, ensuring a longer service life of the biocatalytic filter and a higher removal efficiency (Table 3). Using the covalently functionalized textile, the PM was able to remove 81%–84% DF in continuous operation. Adsorption of DF on the functionalized textile could not be neglected (Figure S3). Carrying out the reference experiment without enzyme, the adsorbed DF was calculated until the breakthrough or until the point where no more adsorption occurred in the reference experiments. As shown in Table 3, the adsorptive effects accounted for 10 to 15 % of the observed DF removal.

**TABLE 3:** Numerical analysis with Equation (1) of the continuous DF removal using a biocatalytic textile obtained from the indicated immobilization method in the plate module (PM): The breakthrough point (BT) is given in terms of flow volume and operation time to breakthrough. *k* is the slope parameter from the Gompertz function, *removal* depicts DF removal until breakpoint, *adsorption* the DF adsorption until BT in the reference experiment without enzyme, and *N_V_* is the number of column volumes until BT.

Immobilization	Flow rate (mL/min)	*k* (L^−1^)	*BT* (L)	*BT* (h)	Removal (%)	Adsorption (%)	*N_V_*
Adsorptive	5.0	0.60 ± 0.012	1.72 ± 0.021	5.73 ± 0.07	73.7 ± 0.8	15.5 ± 0.3	2.1 ± 0.03
Covalent	2.5	0.19 ± 0.010	5.54 ± 0.120	36.93 ± 0.07	80.5 ± 1.1	10.0 ± 1.1	6.7 ± 0.15
Covalent	5.0	0.23 ± 0.004	4.88 ± 0.040	16.30 ± 0.13	83.9 ± 9.7	10.0 ± 1.1	5.91 ± 0.05

To determine if longer reaction times could improve removal efficiencies, the flow rate was halved, and the resulting breakthrough curve of DF in the PM was analyzed (Figure 6). Interestingly, the initial removal efficiency was not affected by the increased residence time indicating that DF removal was not limited by reaction time. However, more water could be treated until breakthrough at the reduced flow rate. These results indicated that breakthrough was primarily driven by enzyme leaching, as confirmed by the detection of leached enzyme in the effluent (Figure S4). Reduced leaching at lower flow rates (Figure S4) increased the flow volume to breakthrough supporting the conclusion that hydrodynamic shear forces contributed to enzyme leaching. It is important to note that the remaining immobilized laccase on the textile remained active over the course of days, as evidenced by a maximum observed operating time to breakthrough of 37 h. Moreover, the biocatalytic textile remained completely permeable during all experiments of maximum 42 h (WM) and no fouling was observed within max. 73 h of the experiment (PM).

### 3.4 Continuous Chemo‐Enzymatic Degradation of Diclofenac

In a final step, the application of the biocatalytic filter as posttreatment of ozonation products was evaluated. Therefore, a diclofenac solution was ozonated and fed to the plate module equipped with covalently immobilized laccase in a continuous experimental setup.

The ozonated solution contained a variety of transformation products, 14 of which were identified through HPLC‐HRMS analysis, in accordance with our prior analysis [19]. The breakthrough point for the enzymatic filter was defined via the breakthrough of the 10 mg/L diclofenac solution ((4.88 ± 0.04) L). The removal efficiencies of the TPs were determined by comparing their relative HPLC‐HRMS peak areas before and after biocatalytic treatment. The respective removal efficiencies of the transformation products (TPs) until this BT are shown in Table 4. For comparison, the adsorption of these TPs on the enzyme‐free textile was examined (Table 4). The breakthrough curves of all TPs and the respective reference experiments are presented in Figure S5. It can be inferred that certain transformation products, such as TP 167 and TP 252, did not exhibit any adsorptive effects. This suggests that their degradation was solely due to the biocatalytic action of the immobilized laccase. Although TP 167′, TP 176, TP 276, TP 290, TP 310, TP 306, TP 306′ and TP 325 initially exhibited adsorption effects, the influence of these effects was ultimately outweighed by that of the biocatalytic degradation process. On the other hand, TP 279, TP 279′, TP 279″, and TP 299 exhibited degradation primarily attributable to adsorption, as their degradation curves aligned with those of adsorption. These transformation products displayed a range of breakthrough behaviors, varying from robust degradation throughout the experiment (e.g., TP 167′) to slow (e.g., TP 325) or early breakthrough (e.g., TP 250) (Table S3).

**TABLE 4:** Ozonation products of diclofenac and their removal efficiency by the biocatalytic PM (biocatalytic removal) and the enzyme‐free textile in the PM (adsorption), respectively, until the breakthrough of 10 mg/L diclofenac (4.88 ± 0.040 L). Those removal efficiencies that were predominantly affected by enzymatic degradation are shown in bold numbers, removal efficiencies resulting from adsorption are shown in italic numbers.

Designation	Proposed structure	Biocatalytic removal (%)	Adsorption (%)
TP 167	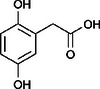	**65.6 ± 0.3**	—
TP 167′	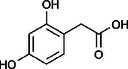	**97.9 ± 0.05**	58.4 ± 0.09
TP 176	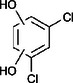	**91.5 ± 0.3**	39.7 ± 0.6
TP 252	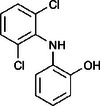	17.3 ± 2.0	—
TP 276	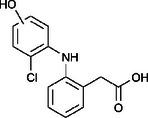	**41.3 ± 0.6**	**25.2 ± 2.8**
TP 279	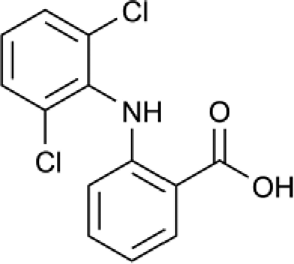	*84.7* ± *0.3*	*83.6* ± *3.0*
TP 279′	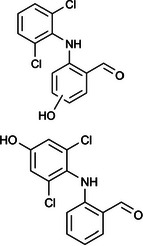	*76.4* ± *0.5*	*71.2* ± *4.0*
TP 279″	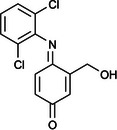	*77.4* ± *0.5*	*76.2* ± *0.03*
TP 290	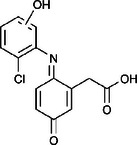	**78.9 ± 0.2**	63.3 ± 1.2
TP 299	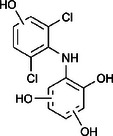	13.6 ± 2.5	6.3 ± 0.6
TP 306	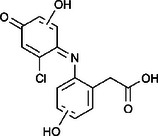	**85.9 ± 0.5**	56.9 ± 2.3
TP 306′	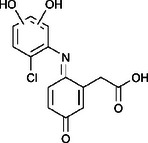	**58.5 ± 2.04**	—
TP 310	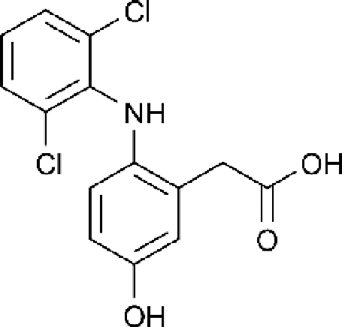	**57.5 ± 0.5**	34.3 ± 0.1
TP 325	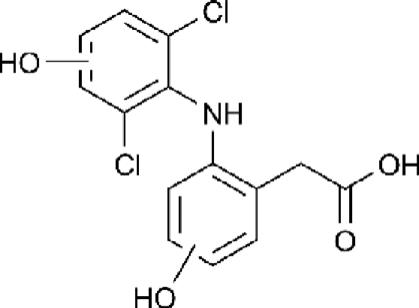	**74.4 ± 0.3**	44.8 ± 0.3

**FIGURE 7:**
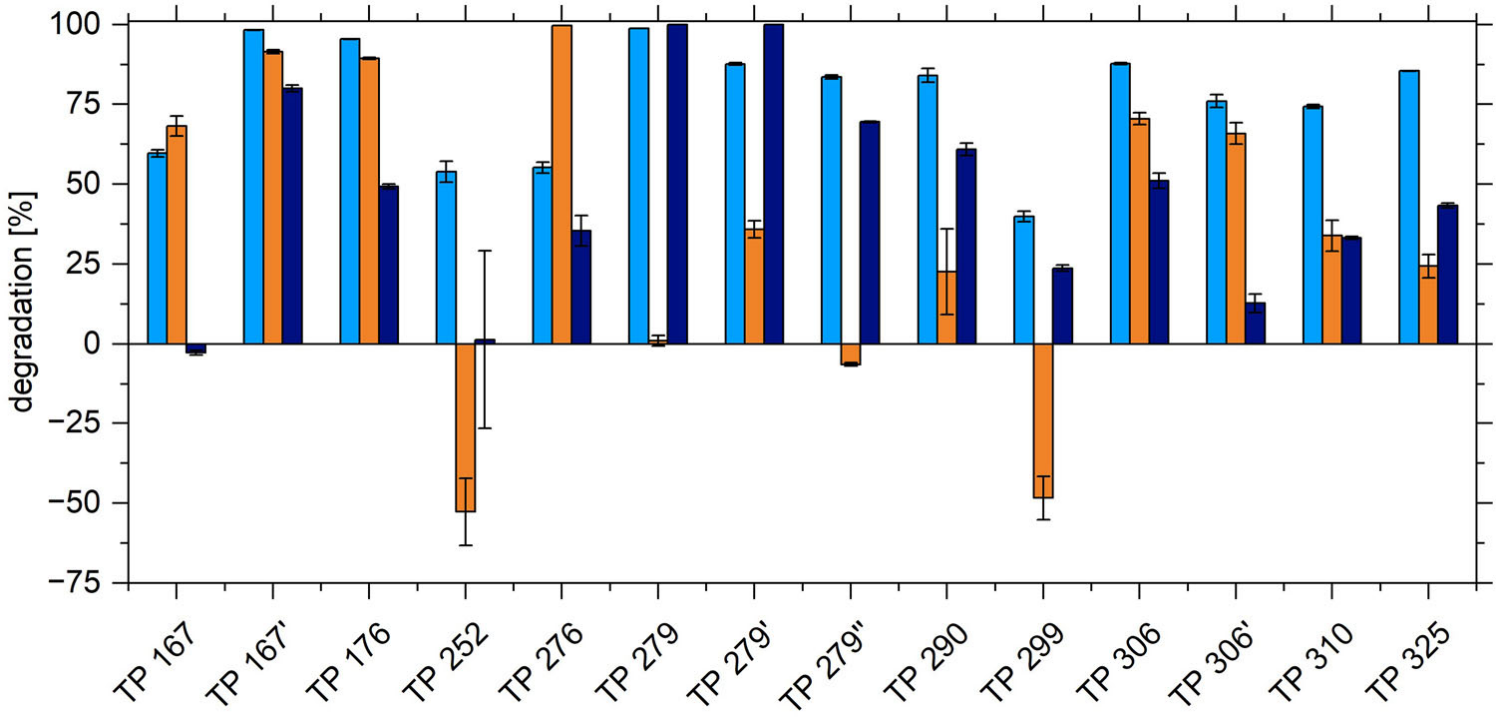
Comparison of the removal efficiencies of ozonation products in the continuous mode in the biocatalytic filter (light blue), and the adsorption (dark blue) with the degradation in the batch process by laccase in solution (orange). The degradation in continuous operation is compared with the degradation in batch operation after 3 h, as the residence time in continuous operation is approximately 2.75 h. Batch mode conditions: 250 U/L ± 10 U/L, 20°C, 100 rpm, continuous mode conditions: room temperature, 343 U/L ± 38 U/L.

In Figure 7, the removal efficiencies of the ozonation products in the presented continuous process with the biocatalytic filter is juxtaposed to the degradation effected with laccase in solution in a batch process as performed earlier [19]. The continuous process nearly universally yielded higher degradation efficiencies than the batch process. In some cases, this was assigned to adsorption effect. Interestingly, the quinoide products as TP 290 and TP 306 showed improved degradation efficiencies in the continuous process. This was attributed to the presence of a dynamic equilibrium in the continuous flow. In the batch process, a thermodynamic equilibrium of coupled redox pairs was established, hindering efficient degradation [19].

Moreover, the formation of TP 252 and TP 299 was not observed in the continuous process. These TPs result from hydroxylation reactions, a reaction which can occur either chemically by ozonation or enzymatically. As illustrated in Figure 7, the continuous process facilitated a faster further reaction with partners potentially accumulated in the vicinity of the biocatalytic textile. This comparison indicates that the biocatalytic filter not only offers an economical setup, enabling the continuous reuse of the enzyme as a heterogeneous textile biocatalyst, but also achieves more efficient TP degradation due to its continuous operation mode.

## 4 Concluding Remarks

A wastewater treatment module for the continuous biocatalytic treatment of micropollutants and their ozonation products was designed on the laboratory scale based on a textile equipped with laccase from *T. versicolor* as heterogeneous biocatalyst. Therefore, the laccase was immobilized on a polyester felt via two different methods, adsorption, and covalent immobilization by an isocyanate crosslinker. The treatment of model wastewater containing the dye RBB showed that the covalent immobilization of the laccase was essential for a continuous operation. As the optimized module was successfully used to post‐treat the ozonation process of diclofenac on a laboratory scale, we suggest that the developed biocatalytic filter design is a promising method to remove potentially harmful ozonation products of micropollutants in a continuous process. While the present work demonstrates the feasibility and advantages of an enzymatic textile filter for advanced wastewater treatment, further work will be required to increase the irreversibility and density of the covalent enzyme loading of the textile in preparation for technical application.

## Author Contributions

**Dorothee Schmiemann:** investigation, formal analysis, writing–original draft, writing–review and editing, visualization. **Jessica Schneider:** investigation, formal analysis. **Marcel Remek:** investigation. **Jeremy Kaulertz:** investigation. **Oliver Seifert:** investigation. **Monika Weidmann:** investigation. **Klaus Opwis:** conceptualization, resources, funding acquisition, writing–review and editing. **Arno Cordes:** conceptualization, resources, funding acquisition, writing–review and editing. **Martin Jäger:** resources, writing–review and editing. **Jochen Stefan Gutmann:** supervision, resources, writing–review and editing. **Kerstin Hoffmann‐Jacobsen:** conceptualization, supervision, resources, funding acquisition, project administration, writing–review and editing.

## Conflicts of Interest

The authors declare no conflicts of interest.

## Supporting information



Supporting information

## Data Availability

The data of this study is available from the corresponding author upon reasonable request.

## References

[1] O. F. S. Khasawneh and P. Palaniandy , “Occurrence and Removal of Pharmaceuticals in Wastewater Treatment Plants,” Process Safety and Environmental Protection 150 (2021): 532–556.

[2] M. Köck‐Schulmeyer , M. Villagrasa , M. López de Alda , et al., “Occurrence and Behavior of Pesticides in Wastewater Treatment Plants and Their Environmental Impact,” The Science of the Total Environment 458‐460 (2013): 466–476. 10.1016/j.scitotenv.2013.04.010 23692851

[3] N. Vieno and M. Sillanpää , “Fate of Diclofenac in Municipal Wastewater Treatment Plant—A Review,” Environment International 69 (2014): 28–39. 24791707 10.1016/j.envint.2014.03.021

[4] X. T. Bui , T. P. T. Vo , H. H. Ngo , et al., “Multicriteria Assessment of Advanced Treatment Technologies for Micropollutants Removal at Large‐Scale Applications,” The Science of the Total Environment 563‐564 (2016): 1050–1067. 10.1016/j.scitotenv.2016.04.191 27198651

[5] G. Crini and E. Lichtfouse , “Advantages and Disadvantages of Techniques Used for Wastewater Treatment,” Environmental Chemistry Letters 17 (2019): 145–155.

[6] M. Voigt , A. Wirtz , K. Hoffmann‐Jacobsen , and M. Jaeger , “Prior Art for the Development of a Fourth Purification Stage in Wastewater Treatment Plant for the Elimination of Anthropogenic Micropollutants‐a Short‐review,” AIMS Environmental Science 7 (2020): 69–98.

[7] I. Alessandretti , C. V. T. Rigueto , M. T. Nazari , et al., “Removal of Diclofenac From Wastewater: A Comprehensive Review of Detection, Characteristics and Tertiary Treatment Techniques,” Journal of Environmental Chemical Engineering 9 (2021): 106743.

[8] M. D. Khan , A. Singh , M. Z. Khan , et al., “Current Perspectives, Recent Advancements, and Efficiencies of Various Dye‐containing Wastewater Treatment Technologies,” Journal of Water Process Engineering 53 (2023): 103579.

[9] Y. Lee and U. V. Gunten , “Advances in Predicting Organic Contaminant Abatement During Ozonation of Municipal Wastewater Effluent: Reaction Kinetics, Transformation Products, and Changes of Biological Effects,” Environmental Science: Water Research & Technology 2 (2016): 421–442.

[10] S. Kharel , M. Stapf , U. Miehe , et al., “Ozone Dose Dependent Formation and Removal of Ozonation Products of Pharmaceuticals in Pilot and Full‐Scale Municipal Wastewater Treatment Plants,” Science of the Total Environment 731 (2020): 139064. 32413657 10.1016/j.scitotenv.2020.139064

[11] S. Svebrant , R. Spörndly , R. H. Lindberg , et al., “On‐Site Pilot Testing of Hospital Wastewater Ozonation to Reduce Pharmaceutical Residues and Antibiotic‐Resistant Bacteria,” Antibiotics (Basel, Switzerland) 10 (2021): 684. 34201188 10.3390/antibiotics10060684 PMC8228021

[12] A. Magdeburg , D. Stalter , M. Schlüsener , et al., “Evaluating the Efficiency of Advanced Wastewater Treatment: Target Analysis of Organic Contaminants and (geno‐)Toxicity Assessment Tell a Different Story,” Water Research 50 (2014): 35–47. 24361518 10.1016/j.watres.2013.11.041

[13] U. V. Gunten , “Oxidation Processes in Water Treatment: Are We on Track?,” Environmental Science & Technology 52 (2018): 5062–5075. 29672032 10.1021/acs.est.8b00586

[14] M. Naghdi , M. Taheran , S. K. Brar , et al., “Removal of Pharmaceutical Compounds in Water and Wastewater Using Fungal Oxidoreductase Enzymes,” Environmental Pollution (Barking, Essex: 1987) 234 (2018): 190–213. 29175684 10.1016/j.envpol.2017.11.060

[15] J. O. Unuofin , A. I. Okoh , and U. U. Nwodo , “Aptitude of Oxidative Enzymes for Treatment of Wastewater Pollutants: A Laccase Perspective,” Molecules (Basel, Switzerland) 24 (2019): 2064. 31151229 10.3390/molecules24112064 PMC6600482

[16] P. Sutaoney , S. Pandya , D. Gajarlwar , et al., “Feasibility and Potential of Laccase‐Based Enzyme in Wastewater Treatment Through Sustainable Approach: A Review,” Environmental Science and Pollution Research International 29 (2022): 86499–86527. 35771325 10.1007/s11356-022-21565-4

[17] A. M. Mayer and R. C. Staples , “Laccase: New Functions for an Old Enzyme,” Phytochemistry 60 (2002): 551–565. 12126701 10.1016/s0031-9422(02)00171-1

[18] H. Catherine , M. Penninckx , and D. Frédéric , “Product Formation From Phenolic Compounds Removal by Laccases: A Review,” Environmental Technology & Innovation 5 (2016): 250–266.

[19] D. Schmiemann , F. Bicks , I. Bartels , et al., “Enzymatic Degradability of Diclofenac Ozonation Products: A Mechanistic Analysis,” Chemosphere 358 (2024): 142112. 38677613 10.1016/j.chemosphere.2024.142112

[20] W. Zhou , W. Zhang , and Y. Cai , “Laccase Immobilization for Water Purification: A Comprehensive Review,” Chemical Engineering Journal 403 (2021): 126272.

[21] M. Fernández‐Fernández , M. Á. Sanromán , and D. Moldes , “Recent Developments and Applications of Immobilized Laccase,” Biotechnology Advances 31 (2013): 1808–1825. 22398306 10.1016/j.biotechadv.2012.02.013

[22] S. Datta , R. Veena , M. S. Samuel , and E. Selvarajan , “Immobilization of Laccases and Applications for the Detection and Remediation of Pollutants: A Review,” Environmental Chemistry Letters 19 (2021): 521–538.

[23] D. S. Wunschik , A. Lorenz , K. N. Ingenbosch , et al., “Activation and Stabilization of Lipase B From *Candida antarctica* by Immobilization on Polymer Brushes With Optimized Surface Structure,” Applied Biochemistry and Biotechnology 194 (2022): 3384–3399. 35357660 10.1007/s12010-022-03913-9 PMC9270307

[24] R. Plagemann , J. Langermann , and U. von Kragl , “Microwave‐Assisted Covalent Immobilization of Enzymes on Inorganic Surfaces,” Engineering in Life Sciences 14 (2014): 493–499.

[25] K. Kiehl , K. Opwis , and J. S. Gutmann , “Polyvinylamine‐Coated Polyester Fibers as a Carrier Matrix for the Immobilization of Peroxidases,” Engineering in Life Sciences 17 (2017): 645–652. 32624810 10.1002/elsc.201600170 PMC6999199

[26] K. Courth , M. Binsch , W. Ali , et al., “Immobilization of Peroxidase on Textile Carrier Materials and Their Application in the Bleaching of Colored Whey,” Journal of Dairy Science 104 (2021): 1548–1559. 33309341 10.3168/jds.2019-17110

[27] M. N. Morshed , N. Behary , N. Bouazizi , et al., “An Overview on Biocatalysts Immobilization on Textiles: Preparation, Progress and Application in Wastewater Treatment,” Chemosphere 279 (2021): 130481. 33894516 10.1016/j.chemosphere.2021.130481

[28] J. Zdarta , K. Jankowska , M. Wyszowska , et al., “Robust Biodegradation of Naproxen and Diclofenac by Laccase Immobilized Using Electrospun Nanofibers With Enhanced Stability and Reusability,” Materials Science & Engineering C, Materials for Biological Applications 103 (2019): 109789. 31349507 10.1016/j.msec.2019.109789

[29] R. Xu , R. Tang , Q. Zhou , et al., “Enhancement of Catalytic Activity of Immobilized Laccase for Diclofenac Biodegradation by Carbon Nanotubes,” Chemical Engineering Journal 262 (2015): 88–95.

[30] M. Taheran , M. Naghdi , S. K. Brar , et al., “Covalent Immobilization of Laccase Onto Nanofibrous Membrane for Degradation of Pharmaceutical Residues in Water,” ACS Sustainable Chemistry & Engineering 5 (2017): 10430–10438.

[31] M. Primožič , G. Kravanja , Ž. Knez , et al., “Immobilized Laccase in the Form of (magnetic) Cross‐Linked Enzyme Aggregates for Sustainable Diclofenac (bio)Degradation,” Journal of Cleaner Production 275 (2020): 124121.

[32] M. Masjoudi , M. Golgoli , Z. Ghobadi Nejad , et al., “Pharmaceuticals Removal by Immobilized Laccase on Polyvinylidene Fluoride Nanocomposite With Multi‐Walled Carbon Nanotubes,” Chemosphere 263 (2021): 128043. 33297058 10.1016/j.chemosphere.2020.128043

[33] M. Maryšková , M. Schaabová , H. Tománková , et al., “Wastewater Treatment by Novel Polyamide/Polyethylenimine Nanofibers With Immobilized Laccase,” Water 12 (2020): 588.

[34] L. Lonappan , Y. Liu , T. Rouissi , et al., “Adsorptive Immobilization of Agro‐industrially Produced Crude Laccase on Various Micro‐Biochars and Degradation of Diclofenac,” Science of the Total Environment 640‐641 (2018): 1251–1258. 10.1016/j.scitotenv.2018.06.005 30021290

[35] L. N. Nguyen , F. I. Hai , A. Dosseto , et al., “Continuous Adsorption and Biotransformation of Micropollutants by Granular Activated Carbon‐Bound Laccase in a Packed‐Bed Enzyme Reactor,” Bioresource Technology 210 (2016): 108–116. 26803903 10.1016/j.biortech.2016.01.014

[36] J. F. Osma , J. L. Toca‐Herrera , and S. Rodríguez‐Couto , “Transformation Pathway of Remazol Brilliant Blue R by Immobilised Laccase,” Bioresource Technology 101 (2010): 8509–8514. 20609582 10.1016/j.biortech.2010.06.074

[37] H. Xu , G. Boeuf , K. Zhu , et al., “Laccase Cross‐Linked Ultraporous Aluminas for Sustainable Biodegradation of Remazol Brilliant Blue R,” Catalysts 12 (2022): 744.

[38] P. M. Dos Santos , J. R. Baruque , R. K. De Souza Lira , et al., “Corn Cob as a Green Support for Laccase Immobilization‐Application on Decolorization of Remazol Brilliant Blue R,” International Journal of Molecular Sciences 23 (2022):9363. 36012620 10.3390/ijms23169363 PMC9409158

[39] E. N. Prasetyo , S. Semlitsch , G. S. Nyanhongo , et al., “Laccase Oxidation and Removal of Toxicants Released During Combustion Processes,” Chemosphere 144 (2016): 652–660. 26408262 10.1016/j.chemosphere.2015.07.082

[40] L. N. Nguyen , F. I. Hai , W. E. Price , et al., “Continuous Biotransformation of Bisphenol A and Diclofenac by Laccase in an Enzymatic Membrane Reactor,” International Biodeterioration & Biodegradation 95 (2014): 25–32.

[41] L. N. Nguyen , F. I. Hai , W. E. Price , et al., “Degradation of a Broad Spectrum of Trace Organic Contaminants by an Enzymatic Membrane Reactor: Complementary Role of Membrane Retention and Enzymatic Degradation,” International Biodeterioration & Biodegradation 99 (2015): 115–122.

[42] V. Hahn , M. Meister , S. Hussy , et al., “Enhanced Laccase‐Mediated Transformation of Diclofenac and Flufenamic Acid in the Presence of Bisphenol A and Testing of an Enzymatic Membrane Reactor,” AMB Express 8 (2018): 28. 29478084 10.1186/s13568-018-0546-y PMC6890904

[43] S. Yang , F. I. Hai , L. D. Nghiem , et al., “Removal of Bisphenol A and Diclofenac by a Novel Fungal Membrane Bioreactor Operated Under Non‐Sterile Conditions,” International Biodeterioration & Biodegradation 85 (2013): 483–490.

[44] D. Schmiemann , L. Hohenschon , I. Bartels , et al., “Enzymatic Post‐Treatment of Ozonation: Laccase‐Mediated Removal of the By‐Products of Acetaminophen Ozonation,” Environmental Science and Pollution Research International 30 (2023): 53128–53139. 36853537 10.1007/s11356-023-25913-w PMC10119220

[45] M. Stieß , Mechanische Verfahrenstechnik (Springer Berlin Heidelberg, 1997).

[46] K. H. Chu , “Fitting the Gompertz Equation to Asymmetric Breakthrough Curves,” Journal of Environmental Chemical Engineering 8 (2020): 103713.

