# Supplementary material for: Design of a Biocatalytic Filter for the Degradation of Diclofenac and Its Ozonation Products
Source: Eng Life Sci. 2025 May 2;25(5):e70024. doi: 10.1002/elsc.70024 (PMC12048198; doi:10.1002/elsc.70024)
Supplement: Supplementary file 1 — Supporting information [file ELSC-25-e70024-s001.pdf]

Design of a biocatalytic filter for the degradation of diclofenac and its ozonation products

Dorothee Schmiemann[1,2], Jessica Schneider[2,3], Marcel Remek[3], Jeremy Kaulertz[1], Oliver Seifert[1], Monika Weidmann[1], Klaus Opwis[3], Arno Cordes[4], Martin Jäger[1], Jochen Stefan Gutmann[2,3] and Kerstin Hoffmann-Jacobsen[1]

[1]Department of Chemistry and Institute for Coatings and Surface Chemistry, Niederrhein University of Applied Sciences, Adlerstr. 32, 47798 Krefeld, Germany

[2] Institute of Physical Chemistry and CENIDE (Center for Nanointegration), University Duisburg-Essen, Universitätsstraße 5, 45141 Essen, Germany

[3] Deutsches Textilforschungszentrum Nord-West gGmbH, Adlerstr. 1, 47798 Krefeld, Germany

[4]ASA Spezialenzyme GmbH, Am Exer 19c, 38302 Wolfenbüttel, Germany

Correspondence: Kerstin Hoffmann-Jacobsen, kerstin.hoffmann-jacobsen@hs-niederrhein.de, Tel. +49 2151 822 4191, Department of Chemistry and Institute for Coatings and Surface Chemistry, Niederrhein University of Applied Sciences, Adlerstr. 32, 47798 Krefeld, Germany

1 Characterization of the biocatalytic textiles

Immobilization-method	Before degradation experiments	After degradation experiments		
adsorptive	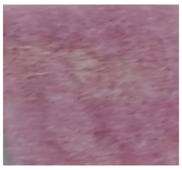	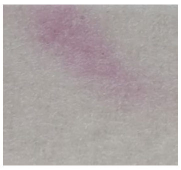	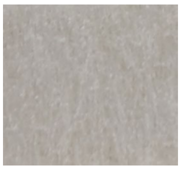	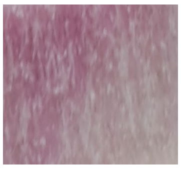
covalent	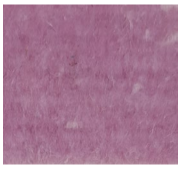	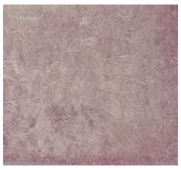	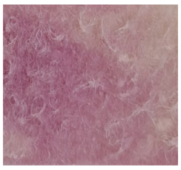	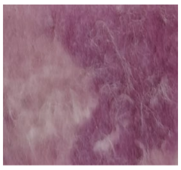

Fig. S1 Photos of the PET textiles (10 x 10 mm) with adsorptively and covalently immobilized enzyme after syringe aldazine activity analysis. The pink color results from the adsorption of tetra-methoxy-azo-bismethylene quinone (oxidized syringaldazine). Textile samples were taken before and after the degradation experiments of diclofenac in the plate module. Due to the coverage inhomogeneity after the experiment, three different sections of the textile are shown exemplarily.

At the beginning of the tests, the textiles showed a strong adsorption of tetra-methoxy-azo-bis-methylene quinone with both immobilization methods. After the tests, pink color of the adsorptive immobilized textile was significantly less pronounced than with covalent immobilization. Thus, it was assumed that there was more residual laccase activity on the covalently immobilized textile. Furthermore, the photos showed that the residual enzymes were not homogeneously distributed on the textile surface.

2 Degradation of RBB with adsorptive and covalent immobilized laccase in the two filter designs

2.1 Adsorption of RBB on the textile

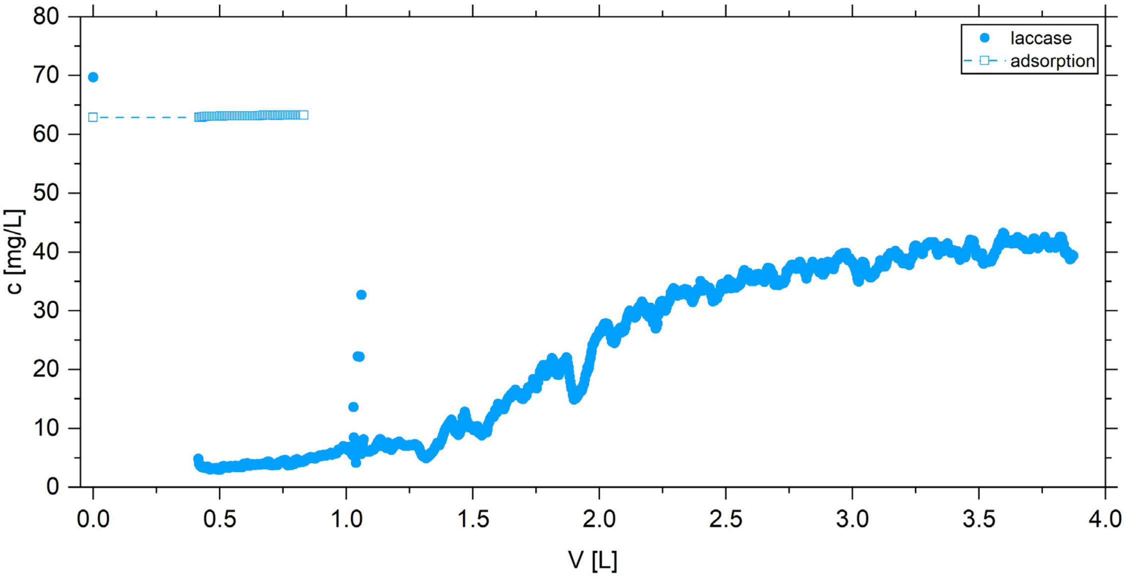

Fig. S2 Breakthrough curves using the reference textiles without enzyme (open squares, adsorption) in comparison to the breakthrough curves with laccase adsorptive immobilized on the textile. Analysis was performed in the winding module (WM) at room temperature.

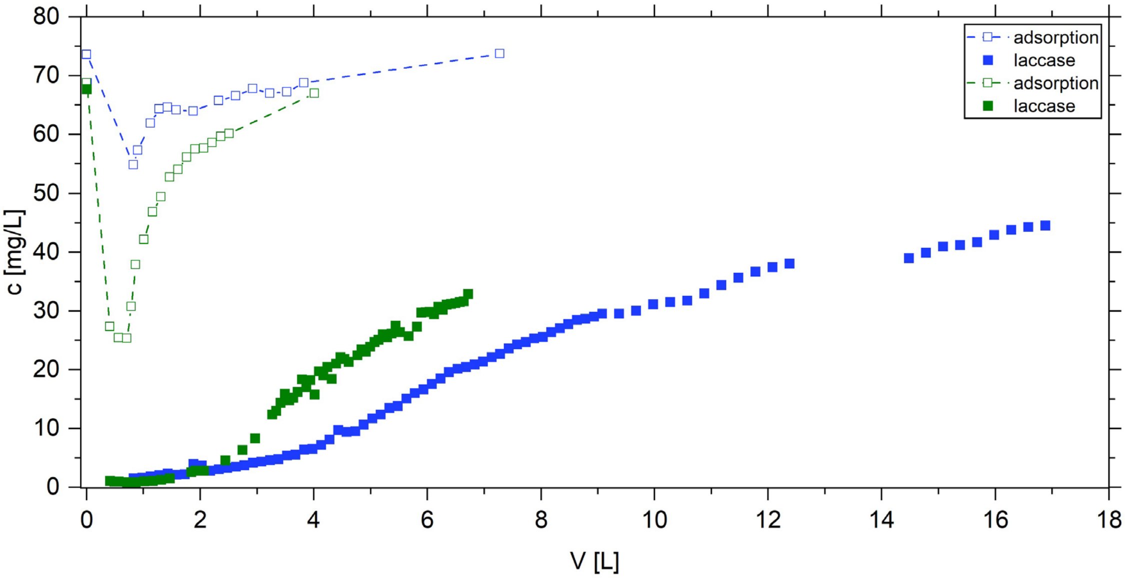

Fig. S3 Breakthrough curves using the reference textiles without enzyme (open squares, adsorption) in comparison to the breakthrough curves with laccase covalently immobilized on the textile. Analysis was performed in the plate module (blue) and in the winding module (green) at room temperature.

The parameters of fit to the RBB breakthrough curves of biocatalytic degradation with Eq. (1) are summarized in Tab. S1.

Tab. S1 Parameters of the fit of Eq. (1) to the breakthrough curves of the degradation of RBB by adsorptive and covalently immobilized laccase in the plate (PM) and winding module (WM): initial concentration (a), breakthrough point (BT), slope parameter *k* from the Gompertz function, and the coefficient of determination (R^2).

	Immobilization	a / mg·L^{-1}	BT / L	k / L^{-1}	R^2
WM	adsorptive	44.05 ± 0.22	1.64 ± 0.006	1.42 ± 0.019	0.98
	covalent	36.83 ± 0.98	3.45 ± 0.056	0.58 ± 0.03	0.99
PM	adsorptive	59.61 ± 0.17	4.88 ± 0.010	0.44 ± 0.002	1.00
	covalent	44.98 ± 0.48	5.99 ± 0.060	0.27 ± 0.006	1.00

3 Degradation of diclofenac in the plate module

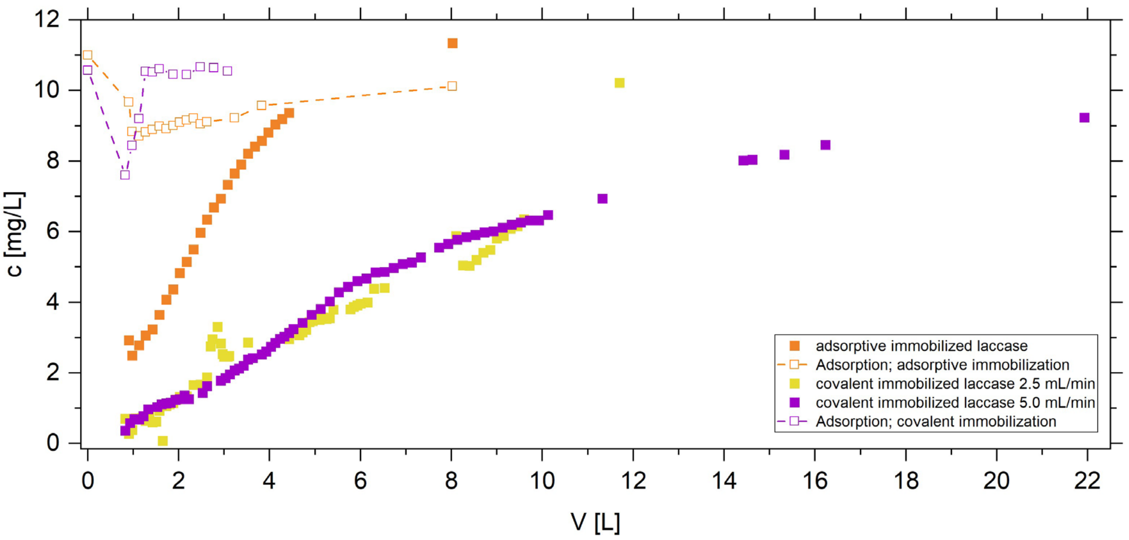

Fig. S3 Breakthrough curves of DF using the biocatalytic textile prepared with the indicated immobilization method and adsorption of DF (open squares) using the respective reference textile without enzyme in the plate module at room temperature.

The parameters of fit of Eq. (1) to the diclofenac breakthrough curves are summarized in Tab. S2.

Tab. S2 Parameters of the fit of Eq. (1) to the breakthrough curves of the degradation of diclofenac by adsorptive and covalently immobilized laccase in the plate (PM) and winding module (WM): initial concentration (a), breakthrough point (BT), slope parameter *k* from the Gompertz function, and the coefficient of determination (R^2).

Immobilization	Flow rate / $mL \cdot min^{-1}$	a / $mg \cdot L^{-1}$	BT / L	k / L^{-1}	R^2
adsorptive	5	11.34 ± 0	1.72 ± 0.021	0.60 ± 0.012	0.99
covalent	2.5	10.22 ± 0	5.54 ± 0.120	0.19 ± 0.010	0.92
covalent	5	9.24 ± 0	4.88 ± 0.040	0.23 ± 0-004	0.99

4 Leaching of covalently immobilized laccase at different flow rates in the plate module

The laccase activity in the effluent of the plate module was analyzed during DF treatment covalently immobilized laccase at different flow rates in the plate module (Fig. S4).

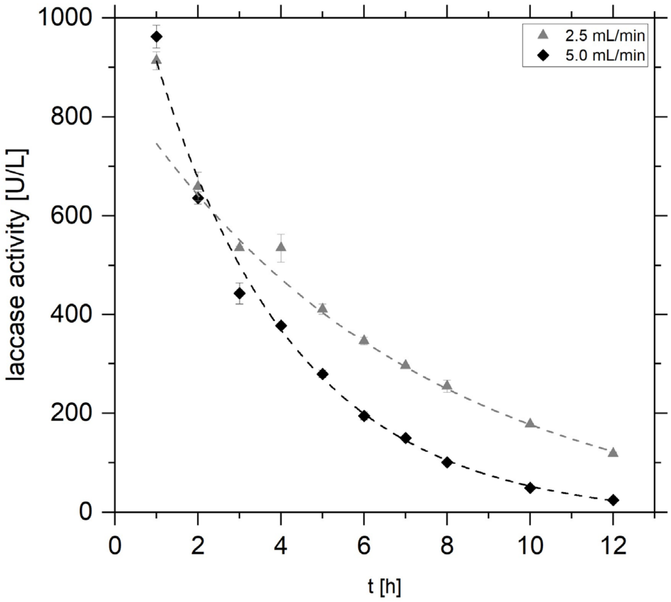

Fig. S4 Leaching of covalently immobilized laccase at different flow rates in the plate module. The dashed line represents a single-exponential fit.

5 Degradation of ozonated DF with covalently immobilized laccase

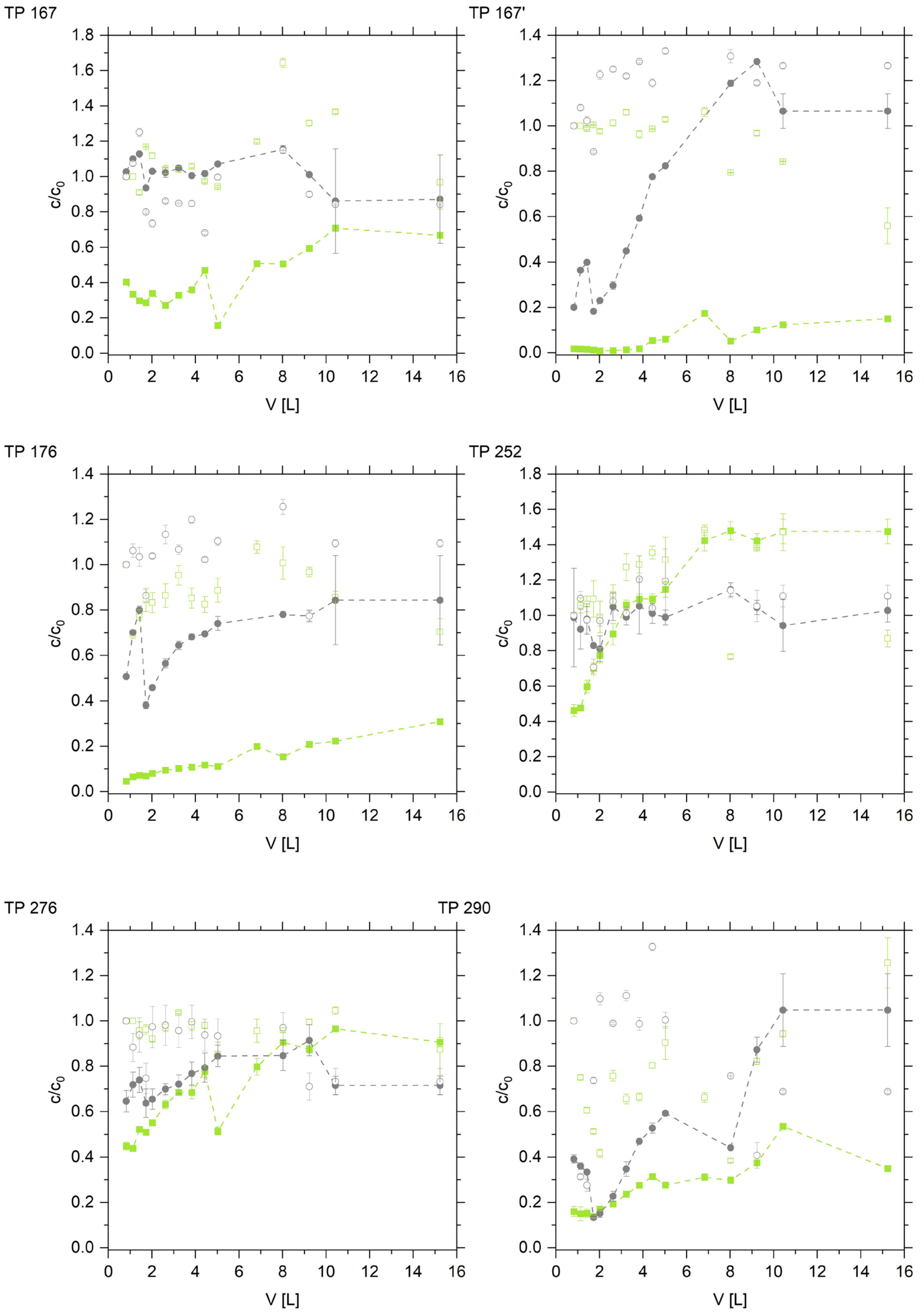

TP 279

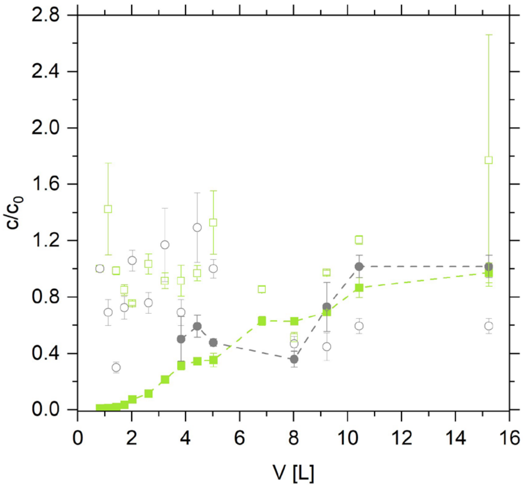

TP 279'

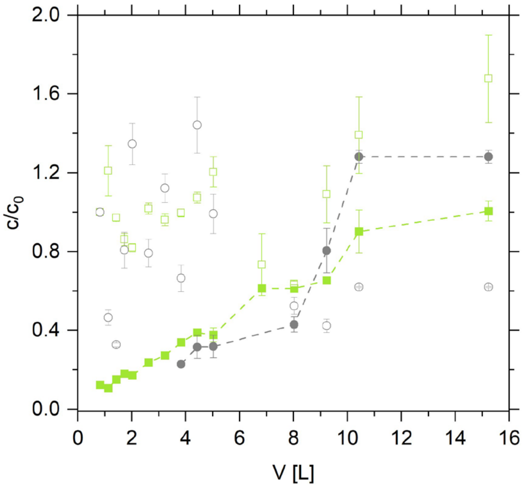

TP 279''

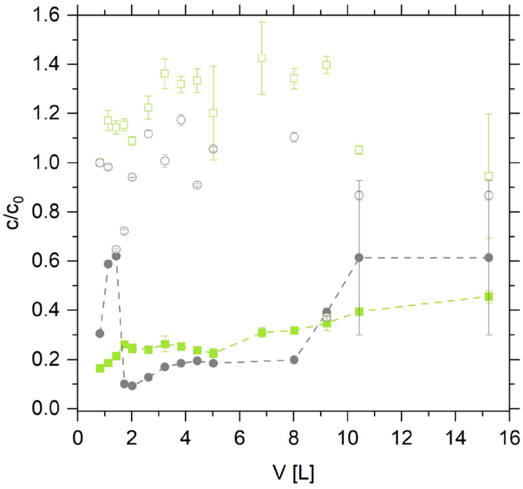

TP 299

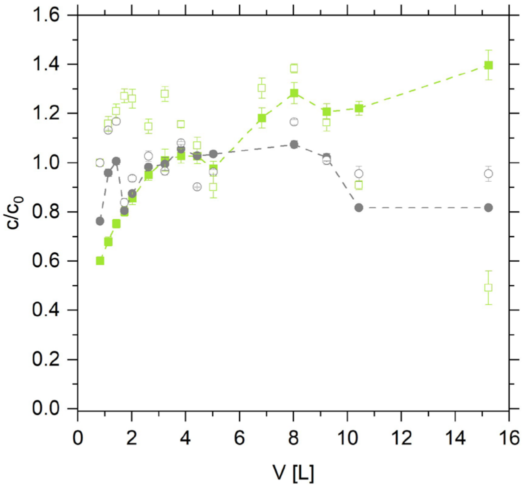

TP 306

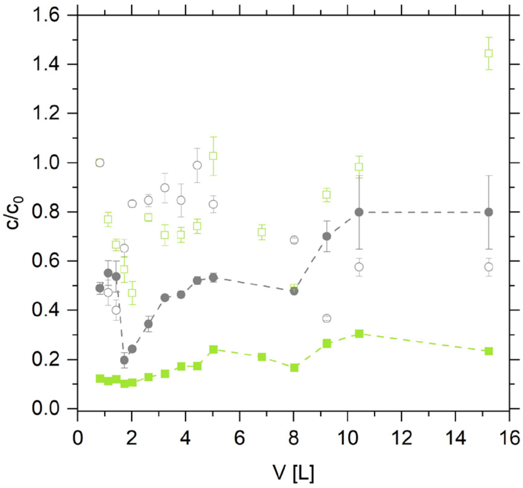

TP 306'

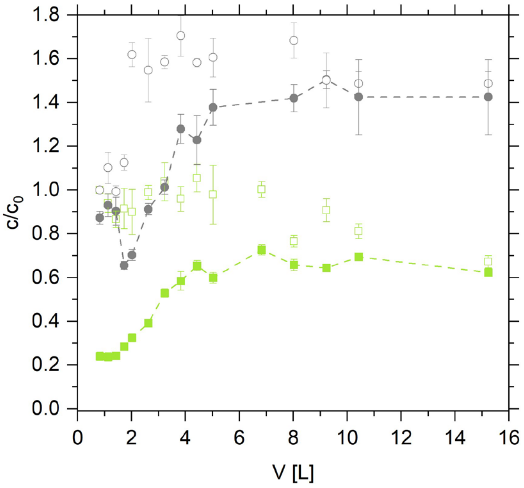

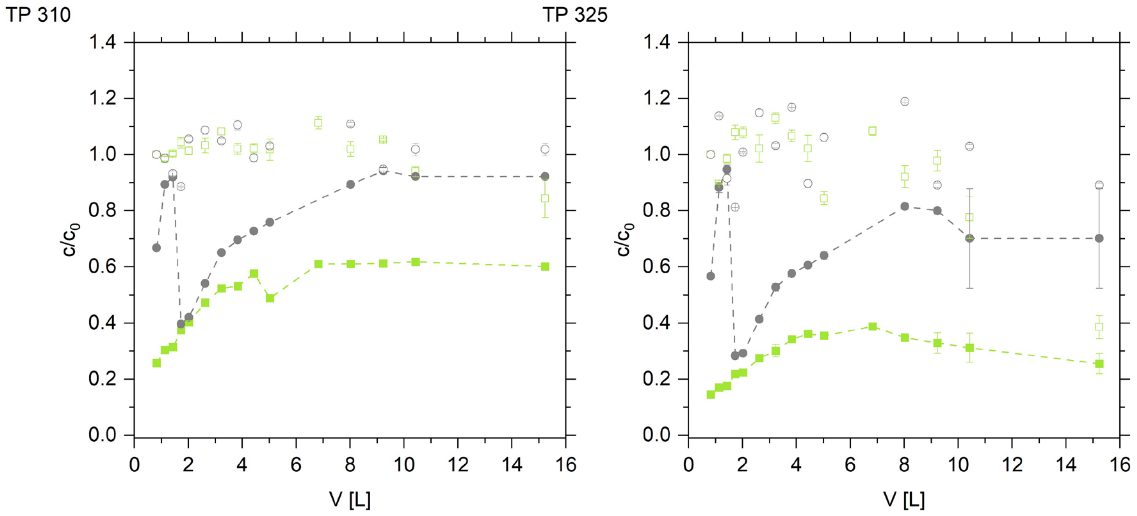

Fig. S5 Degradation of diclofenac transformation products by immobilised laccase from T. versicolor in continuous mode in the plate module. The filled green squares indicate the biocatalytic degradation, while the unfilled green squares indicate the reference (feed). The filled grey circles show the adsorption on the covalent PET textile without enzyme, the unfilled grey circles show the reference (feed). The kinetic measurements by LC-MS were carried out in triplicate ($\bar{x} \pm$ SD).

The reference fluctuations occur because the ozonation was performed discontinuously and new ozonated solution was added to the feed reservoir every 2-3 hours.

Tab. S3 Breakthrough points of the individual ozonation products determined by the fitting of Eq. (1) to the breakthrough curves depicted in Fig. S 5. The amplitude was set to 1 for all fits. The biocatalytic degradation efficiency (degradation) and the adsorption on the textile without enzyme (adsorption) was calculated as integral to the individual BT.

Designation	BT	degradation	adsorption
	L	%	%
TP 167	-[1]	-[1]	-[1]
TP 167‘	15.77 ± 1.14		
	R^2 0.84	91.23 ± 0.07	13.79 ± 2.89
TP 176	14.23 ± 1.04		
	R^2 0.88	82.51 ± 0.69	25.94 ± 6.42
TP 252	0.87 ± 0.22		-[2]
	R^2 0.73	53.88 ± 3.30	

TP 276	0.20 ± 0.26	-[3]	-[3]
	R^2 0.92		
TP 290	10.65 ± 1.94		
	R^2 0.46	72.68 ± 0.47	48.89 ± 0.10
TP 279	9.51 ± 1.66		
	R^2 0.59	60.33 ± 0.74	69.03 ± 3.00
TP 279‘	4.97 ± 0.27		
	R^2 0.93	76.36 ± 0.50	89.70 ± 1.42
TP 279‘‘	3.57 ± 0.45		
	R^2 0.98	77.84 ± 0.94	74.72 ± 0.14
TP 299	0.23 ± 0.34	-[3]	-[3]
	R^2 0.77		
TP 306	-[1]	-[1]	-[1]
TP 306‘	2.45 ± 0.66		
	R^2 0.77	72.35 ± 1.62	18.25 ± 0.35
TP 310	1.62 ± 0.16		
	R^2 0.77	71.49 ± 0.30	27.25 ± 0.31
TP 325	6.85 ± 0.74		
	R^2 0.79	71.37 ± 0.31	38.09 ± 0.43

1) The fit did not converge.
2) No adsorption
3) The calculated breakthrough and the error are identical: no degradation.
